# Rapid Detection of *Salmonella typhimurium* in Drinking Water by a White Light Reflectance Spectroscopy Immunosensor

**DOI:** 10.3390/s21082683

**Published:** 2021-04-10

**Authors:** Michailia Angelopoulou, Konstantina Tzialla, Angeliki Voulgari, Mary Dikeoulia, Ioannis Raptis, Sotirios Elias Kakabakos, Panagiota Petrou

**Affiliations:** 1 Immunoassays/Immunosensors Lab, Institute of Nuclear & Radiological Sciences & Technology, Energy & Safety, National Centre for Scientific Research “Demokritos”, 15341 Aghia Paraskevi, Greece; konstantina.tz94@gmail.com (K.T.); skakab@rrp.demokritos.gr (S.E.K.); 2 Delta Foods S.A., 14565 Agios Stefanos, Greece; aggbou@delta.gr (A.V.); mardik@delta.gr (M.D.); 3 ThetaMetrisis S.A., 12132 Egaleo, Greece; raptis@thetametrisis.com

**Keywords:** bacteria, *Salmonella typhimurium*, white light reflectance spectroscopy, immunosensor

## Abstract

Biosensors represent an attractive approach for fast bacteria detection. Here, we present an optical biosensor for the detection of *Salmonella typhimurium* lipopolysaccharide (LPS) and *Salmonella* bacteria in drinking water, based on white light reflectance spectroscopy. The sensor chip consisted of a Si die with a thin SiO_2_ layer on top that was transformed into a biosensor through the immobilization of *Salmonella* LPS. The optical setup included a reflection probe with seven 200 μm fibers, a visible and near-infrared light source, and a spectrometer. The six fibers at the reflection probe circumference were coupled with the light source and illuminated the biosensor chip vertically, whereas the central fiber collected the reflected light and guided it to the spectrometer. A competitive immunoassay configuration was adopted for the analysis. Accordingly, a mixture of LPS or bacteria solution, pre-incubated for 15 min, with an anti-*Salmonella* LPS antibody was pumped over the chip followed by biotinylated secondary antibody and streptavidin for signal enhancement. The binding of the free anti-*Salmonella* antibody to chip-immobilized LPS led to a shift of the reflectance spectrum that was inversely related to the analyte concentration (LPS or bacteria) in the calibrators or samples. The total assay duration was 15 min, and the detection limits achieved were 4 ng/mL for LPS and 320 CFU/mL for bacteria. Taking into account the low detection limits, the short analysis time, and the small size of the chip and instrumentation employed, the proposed immunosensor could find wide application for bacteria detection in drinking water.

## 1. Introduction

Foodborne diseases caused by the consumption of food or water contaminated with bacteria are a serious public health issue worldwide [1]. According to the World Health Organization, approximately 5 million deaths annually, mainly in developing countries, are attributed to contaminated water [2]. The problem, however, is global; in the United States in 2017, 841 foodborne disease outbreaks were reported by public health authorities from 50 states, resulting in 14,481 illnesses, 827 hospitalizations, and 20 deaths [3]. The bacteria responsible for these outbreaks are mainly those belonging to species of *Salmonella*, *Escherichia coli*, *Vibrio cholera, Listeria monocytogenes*, and *Shigella* [1,2]. Among them, *Salmonella* is the pathogen most frequently responsible for foodborne illnesses worldwide [3,4]. *Salmonella* is a genus of facultatively anaerobic, rod-shaped, gram-negative bacteria, which belongs to the Enterobacteriaceae family and contains more than 2500 serotypes, from which *Salmonella enterica serovar typhimurium* (*S. typhimurium*) is the one most frequently associated with foodborne illnesses in humans [5]. The consumption of food or water contaminated with *S. typhimurium* causes diarrhea, abdominal pain, nausea, and stomach discomfort, and the clinical symptoms usually last up to 5 days [6]. The wide spread of *Salmonella*, its resistance to drugs, as well as the similarity of salmonellosis symptoms with other diseases, makes difficult the effective management of disease outbreaks caused by this bacterium. Thus, the only way to prevent the outbreaks is to test food and water for the presence of *Salmonella* prior to their release for consumption. In this frame, regulatory authorities worldwide have issued regulations regarding the presence and detection of bacteria in foodstuffs. For example, EU regulations require the absence of *Salmonella* in 25 g of food samples (CE 1441/2007), including not only water but also meat and its products, dairy products, eggs and egg-containing foods, crustaceans, and molluscan shellfish, etc. [7].

The methods currently employed for the detection and identification of bacteria in food and water are based on microbiological methods such as culturing and plating. These methods are reliable but include several steps such as pre-enrichment, selective enrichment, isolation, and identification of colonies through biochemical and serological tests, which are time-consuming and require at least 5–7 days to complete [8,9]. In order to shorten the analysis time to 8–48 h, DNA-based methods such as PCR, quantitative PCR, and multiplex PCR have been employed for bacteria detection and identification, alleviating the necessity of the selective plating steps [10,11]. A significant drawback of these methods is the inability to discriminate between live and dead bacterial cells, leading frequently to false-positive results [12]. Immunological methods, i.e., enzyme-linked immunosorbent assay (ELISA) and immunochromatographic strips [13,14], are specific and fast methods for bacteria detection; however, they also require an enrichment culture step since their detection sensitivity does not meet the regulatory requirements [15]. To improve the applicability of immunochemical methods in bacteria detection in food samples, they have been combined with a bacterial enrichment step, which includes a short culture followed by analysis and/or concentration of bacteria from large sample volumes via filtration or immunomagnetic separation. Most of the above methods could provide detection limits down to 1–10 CFU/25 g [16]; however, these additional steps prolong the time needed for analysis and make the method incompatible with the turn-around production time and the short shelf-life of products that are not highly pasteurized [17].

For these reasons, biosensors based on electrochemical, piezoelectric, or optical transducers are gaining ground in foodborne bacteria detection. They indeed hold the promise for fast, real-time and sensitive measurements at the point of need. Regarding electrochemical immunosensors, devices employing amperometric, potentiometric, impedimetric, and conductimetric detection principles have been exploited for bacteria detection [18,19,20,21,22]. Especially attractive are label-free electrochemical sensors based on potentiometry or impedance measurements, which achieve detection limits comparable to or lower than those employing labels for detection [4,22,23]. On the other hand, immunosensors based on piezoelectric transducers offer label-free detection but their sensitivity is inadequate for food analysis [24,25]. A great variety of optical biosensors based on different transduction principles (light absorbance, fluorescence, chemiluminescence, light polarization, surface plasmon resonance (SPR), and total internal reflectance) [26,27] have also been implemented for foodborne bacteria detection. Amongst them, commercially available fiber-optic or SPR platforms [28,29,30], such as Biacore and Spreeta, have been used for this purpose. SPR-based sensors display various advantages over other optical methods since they provide for label-free and real-time analysis [16] of single or multiple bacteria in the same sample [31,32]. Although the detection limits for bacteria with SPR sensors are in the order of 10^4^ CFU/mL or higher, there are few reports demonstrating significantly lower detection limits, e.g., 3–20 CFU/mL for *Escherichia coli* and 50 CFU/mL for *Bacillus cereus* [33]. Despite this improvement, most commercially available SPR platforms are bulky and expensive equipment and, therefore, cannot be used at the point of need. On the other hand, optical interferometer sensors offer real-time analysis and have emerged as powerful tools for sensitive multiplexed determination of hazardous substances in food and beverages [34,35,36]. Thus, a Mach–Zehnder interferometer has been used for the immunochemical detection of *E. coli* with a detection limit of 100 CFU/mL [37], and a bi-modal interferometric sensor for the detection of *E. coli* and *Bacillus cereus* with detection limits of 40 CFU/mL for both bacteria [38].

**Figure 1:**
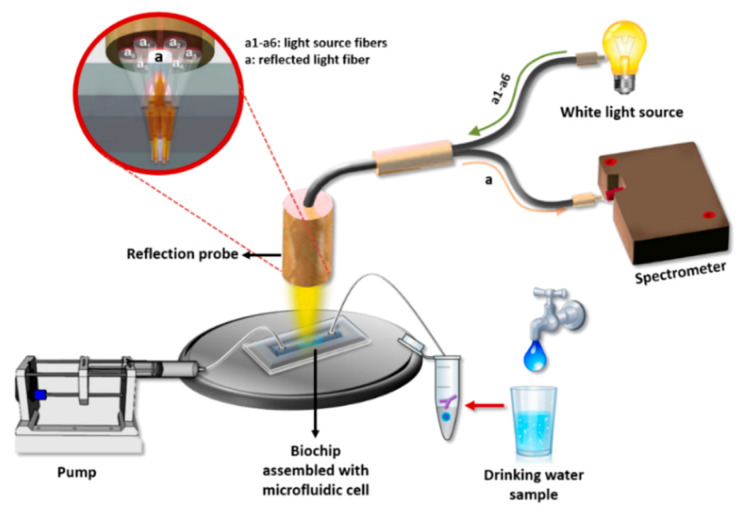
Illustration of the white light reflectance spectroscopy (WLRS) optical setup and sensing principle.

In this work, a label-free immunosensor based on white light reflectance spectroscopy (WLRS) for the rapid determination of *Salmonella* lipopolysaccharide (LPS) as well as *Salmonella* bacteria in drinking water samples is presented for the first time. The WLRS approach has been exploited for the detection of analytes of clinical interest (e.g., C-reactive protein and D-dimer) [39], as well as of analytes related to food safety (e.g., pesticides, mycotoxins) [40,41]. Moreover, it has been shown that it is possible to perform assays with the WLRS setup in complicated matrices such as milk [40] or whole blood [42] by introducing a short washing step after the primary immunoassay step. Thus, although WLRS is less sensitive in terms of refractive index changes detection than other label-free optical techniques such as SPR and might require some signal enhancement steps after immunoreaction, the low costs of instrumentation and related consumables (e.g., chips) render it suitable for the development of devices for on-site analysis. The sensor consists of a Si chip (5 × 15 mm^2^) covered with a thin SiO_2_ layer (approximately 1000 nm) on which the biomolecular reactions take place. The optical setup includes a reflection probe composed of seven 200 μm core fibers. The six fibers, arranged at the circumference of the reflection probe, are coupled to a visible-near infrared (NIR) light source and illuminate the sensor chip vertically through a transparent fluidic compartment, while the seventh fiber, placed at the center of the probe, collects the reflected light and guides it to a visible-NIR spectrometer (Figure 1). When the light strikes the chip, it is partially reflected at each interface, creating an interference spectrum. The buildup of a biomolecular layer on top of the chip, due to biomolecular reactions, increases the optical path length, resulting in a shift of the reflected spectrum that correlates with the concentration of the reacting biomolecules [43]. This spectral shift is transformed online by the dedicated software to increase in thickness, allowing the monitoring of the biomolecular reaction in real-time. For the particular application of *Salmonella* LPS and bacteria detection with the WLRS sensor, a competitive immunoassay was employed by immobilizing *Salmonella* LPS on the WLRS chip. For the assay, mixtures of calibrators/samples with a specific anti-*Salmonella* antibody were run over the chip with the final amount of antibody bound being inversely proportional to the analyte concentration in the calibrator/sample. In order to improve the assay detection limit and keep the assay duration as short as possible, the introduction of signal enhancement steps was investigated. In particular, reaction with biotinylated, anti-species-specific antibody (secondary antibody) followed by reaction with streptavidin was implemented. The shortest duration of each assay step for which adequate signal was achieved was selected for the final protocol. The analytical performance of the WLRS immunosensor was evaluated in terms of sensitivity, reproducibility, water matrix effect, and capability of chip regeneration/reuse. The accuracy of the assay was determined through recovery experiments performed using bottled and tap water, and the assay specificity against other bacteria species was verified. Finally, the performance of the WLRS immunosensor developed, mainly in terms of its detection limit and assay duration, was compared to that of other optical label-free immunosensors reported in the literature for *Salmonella* detection.

## 2. Materials and Methods

### 2.1. Reagents

Petri dishes (92 mm, 16 mm), polystyrene inoculation loops (1 μL), and spreaders were obtained from Sarstedt AG & Co. KG (Numbrecht, Germany). Plate Count Agar (PCA) with skimmed milk was obtained from BIOKAR Diagnostics (Allonne, France). *Salmonella enterica serovar typhimurium* (*S. typhimurium*, ATCC 14028), *Salmonella thomson* and *Escherichia coli* O157:H7 (*E. coli* O157:H7, NCTC 12900) were kindly provided from Delta Foods S.A. (Athens, Greece). Microtiter plates were from Greiner Diagnostic GmbH (Bahlingen, Germany). Lipopolysaccharide (LPS) of *Salmonella enterica serotype typhimurium*, bovine serum albumin (BSA), Goat Anti-Rabbit IgG antibody conjugated with peroxidase, 2,2′-azino-bis (3-ethylbenzthiazoline-6-sulphonic acid) (ABTS), non-labelled Goat Anti-Rabbit IgG antibody, streptavidin, and 3-aminopropyl-triethoxysilane (APTES) were purchased from Sigma-Aldrich (Darmstadt, Germany). Rabbit polyclonal anti-*Salmonella* group antigen antibody (Product number 8209-4006) was from Bio-RAD Abd Serotec Ltd. (Kidlington, Oxford, UK). The antibody was developed using a mixture of *S. typhimurium, S. enteriditis,* and *S. heidelburg* and therefore recognizes *Salmonella* O and H antigens of these serotypes. All other reagents were of analytical grade and were purchased from Sigma-Aldrich. The water used in the study was doubly distilled. Biotinylation of Goat Anti-Rabbit IgG antibody was performed following a previously published protocol [40]. Four-inch Si wafers (100) were obtained from Si-Mat (Kaufering, Germany). Spotting of the chips with the *S. typhimurium* LPS was performed using the BioOdyssey™ Calligrapher MiniArrayer (Bio-Rad Laboratories Inc., Hercules, CA, USA).

### 2.2. WLRS Instrumentation

The WLRS detection system used in this work consists of a visible-near infrared light source (ThetaMetrisis S.A., Athens, Greece), a miniaturized USB controlled spectrometer (Maya 2000 Pro; Ocean Insight, Orlando, FL, USA) with a resolution of 0.25 nm, and a reflection probe (AVANTES Inc., Broomfield, CO, USA) composed of seven optical fibers with a 200 μm diameter each. The six fibers arranged at the circumference of the probe sent the light to the chip surface, whereas the seventh central fiber collected the reflected light and guided it to the spectrometer. For the assay, a fluidic compartment (Jobst Technologies GmbH, Freiburg, Germany) was placed on top of the chip. This compartment was made by attaching a 200 μm thick, double-sided adhesive tape, cut to form a reaction chamber with dimensions 12 mm (L) × 2.5 mm (W) × 0.2 mm (H), to a 2 mm thick poly(methyl methacrylate) (PMMA) cover with drilled fluid inlet and outlet holes. The analysis of the reflected spectrum recorded from the spectrometer (integration time was 15 ms; 1 spectrum per second) was performed by software designed by ThetaMetrisis S.A. that transformed in real-time the spectral shift to effective biomolecular adlayer thickness by applying the Levenberg–Marquart algorithm.

### 2.3. Bacteria Culturing and Counting

Bacteria strains (*S. typhimurium*, *S. thomson,* and *E. coli*) were grown on Petri dishes containing PCA medium through incubation for 18 h at 37 °C. Next, a number of colonies were peaked and suspended in 1 mL phosphate buffer saline (PBS) 10 mM, pH 7.4, and the optical density at 600 nm (OD_600_) was measured using a Novaspec II spectrophotometer (Pharmacia Biotech, UK). The bacteria concentration was determined assuming that OD_600_ = 0.2 corresponds to 3.2 × 10^8^ CFU/mL [7]. The suspension was then diluted to prepare a series of 10-fold dilutions with concentrations down to 10 CFU/mL. To determine the viable bacteria concentration in the suspension, colony counting on PCA Petri dishes inoculated with each one of the suspension dilutions was performed after incubation for 18 h at 37 °C (three plates per dilution).

### 2.4. Calibrators/Water Samples Preparation

A stock solution of *S. typhimurium* LPS with a concentration of 1 mg/mL was prepared in carbonate buffer, pH 9.2 (coating buffer), and stored at 4 °C until use. The stock solution was used for the preparation of calibrators with concentrations ranging from 10 to 1000 ng/mL in 10 mM PBS buffer, pH 7.4, containing 10 g/L BSA (assay buffer). Regarding *S. typhimurium* calibrators, after determining the cell suspension concentration as described in Section 2.3, it was serially diluted to obtain calibrators with concentrations ranging from 5 × 10^2^ to 5 × 10^7^ CFU/mL. For the recovery experiments, 3 bottled, natural mineral waters—namely Zagori (Chitos S.A, Ioannina, Greece), Vikos (Epirotic Bottling Company S.A., Ioannina, Greece), and Avra (Coca-Cola Greek Bottling Company S.A., Maroussi, Greece)—and tap water were spiked with known concentrations of *S. typhimurium* to obtain samples containing 8 × 10^3^ to 8 × 10^5^ CFU/mL.

### 2.5. Preparation and Biofunctionalization of the Chip

A silicon dioxide layer with an average thickness of 1100 nm was grown on the wafers by wet oxidation for 3 h at 1100 °C in the cleanroom facility at the Institute of Nanoscience and Nanotechnology of National Centre for Scientific Research “Demokritos” (Aghia Paraskevi, Greece). Then, the wafers were diced to chips with dimensions 5 × 15 mm. To achieve chemical functionalization of the chips’ surface, cleaning and hydrophilization of the chips were performed by treatment with Piranha solution (1:1 H_2_SO_4_/30% (*v/v*) H_2_O_2_) for 20 min. After intensive washing with distilled water, immersion of the chips in 2% (*v/v*) aqueous APTES solution for 20 min followed. Then, the chips were washed with distilled water, dried under a nitrogen stream, incubated for 20 min at 120 °C, and left at room temperature (RT) for at least 24 h prior to spotting. For the biological activation, a 200 μg/mL LPS solution in 50 mM carbonate buffer, pH 9.2 (coating buffer), was spotted in a 3 × 5 mm^2^ area at the center of the APTES-modified chips using the BioOdyssey™ Calligrapher MiniArrayer, and they were incubated overnight at 4 °C in 75% humidity. Thereafter, a blocking step was performed through the immersion of the chips in a 10 mg/mL BSA solution in 0.1 M NaHCO_3_, pH 8.5 (blocking solution), for 1 h at RT. Finally, the chips were immersed in 10 mM PBS, pH 7.4 (washing buffer A) for 30 s, washed with distilled water, dried under a nitrogen stream, and stored at 4 °C until use.

### 2.6. Competitive Immunodetection of S. typhimurium with the WLRS Chip

**Figure 2:**
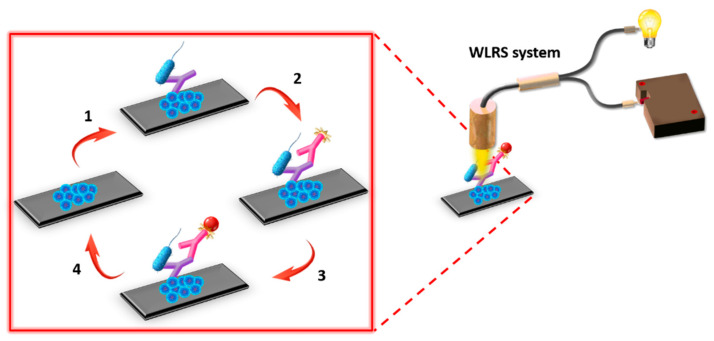
Assay configuration for the detection of *S. typhimurium* employing the WLRS system. The main assay steps included: (1) incubation of immobilized onto the chip lipopolysaccharide (LPS) with a mixture of bacteria calibrator and rabbit polyclonal anti-*Salmonella* antibody, (2) reaction with biotinylated Goat Anti-Rabbit IgG antibody (secondary antibody), (3) reaction with streptavidin, and (4) chip surface regeneration, i.e., removal of the immunosorbed molecules to use the chip for a new assay cycle.

Prior to the assay, the fluidic compartment was assembled with the biofunctionalized chip, and the chip was inserted in the docking station of the apparatus and connected with the pump for the delivery of the reagents. At first, assay buffer was passed over the chip surface to obtain a baseline signal. For the assay, calibrators or water samples were mixed at a 1:1 volume ratio with a 1.5 μg/mL rabbit polyclonal anti-*Salmonella* antibody solution in assay buffer and incubated for 15 min at RT. The mixtures were then pumped over the chip for 7 min with a flow rate of 40 μL/min. Subsequently, a 10 μg/mL biotinylated Goat Anti-Rabbit IgG antibody solution in assay buffer passed for 5 min, followed by a 5 μg/mL streptavidin solution in assay buffer for 3 min. Finally, a regeneration step was performed by running over the chip a 0.1 M HCl solution for 3 min, and equilibration of the chip with assay buffer was carried out prior to the next assay cycle. For each calibrator/sample, the effective biomolecular layer thickness values were determined in nm. Calibration curves were constructed by plotting the percentage of the effective biomolecular layer thickness values obtained for the different calibrators (S_x_) with respect to the value of the zero calibrator (S_0_) against the concentration of LPS or bacteria cells in the calibrators. In Figure 2, a three-dimensional (3-D) scheme of assay configuration for the detection of *S. typhimurium* is presented.

### 2.7. ELISA Method for the Detection of S. typhimurium in Water Samples

One hundred microliters of a 0.5 μg/mL LPS solution in coating buffer were added in 96-well ELISA microtitration plates and incubated overnight at RT. The wells were washed twice with 300 μL of 10 mM PBS buffer, pH 7.4 (washing solution A), and then 300 μL of blocking solution were added. After incubation for 1 h at RT, the wells were washed as previously. Equal volumes of LPS, bacteria calibrators, or samples were mixed with a 100 ng/mL rabbit polyclonal anti-*Salmonella* antibody solution in assay buffer and incubated for 15 min. For the assay, 100 µL from the mixtures were added to wells and were incubated for 1 h under vigorous shaking. After that, the wells were washed four times with 300 μL of washing solution A solution containing 0.05% (*v/v*) Tween 20 (washing solution B), 100 μL of a 10 μg/mL anti-rabbit IgG antibody-horse radish peroxidase (HRP) conjugate solution in 0.1 mM Tris-HCl buffer, pH 8.25, containing 5 mg/mL BSA, and 9 g/L NaCl were added per well and incubated for 40 min under vigorous shaking. The wells were washed as previously, and then they were incubated with 100 μL of HRP substrate solution (0.03% *v/v* H_2_O_2_ and 1.9 µM ABTS in 0.1 M citrate-phosphate buffer, pH 4.5) for 30 min under shaking. The optical density of the wells at 405 nm was measured using a VICTOR3 1420 Multilabel Counter (PerkinElmer). For the calibration curve, the optical density value of the different calibrators (B_x_) expressed as a percentage of the mean zero calibrator optical density value (B_0_) was plotted versus the calibrator concentration.

## 3. Results

### 3.1. Optimization of the Assay

For the detection of LPS or *S. typhimurium* cells with the WLRS sensor, a competitive immunoassay configuration was implemented. In a competitive immunoassay, the maximum signal is obtained in the absence of analyte (zero calibrator), and the signal decreases as the analyte concentration in the calibrators of the samples increases. The percent signal decrease obtained for a given analyte concentration with respect to zero calibrator signal is a measure of the assay sensitivity. Thus, during the development of LPS competitive immunoassay, several parameters have been optimized, taking into account both the maximum signal and the percent signal drop obtained for certain calibrators.

**Figure 3:**
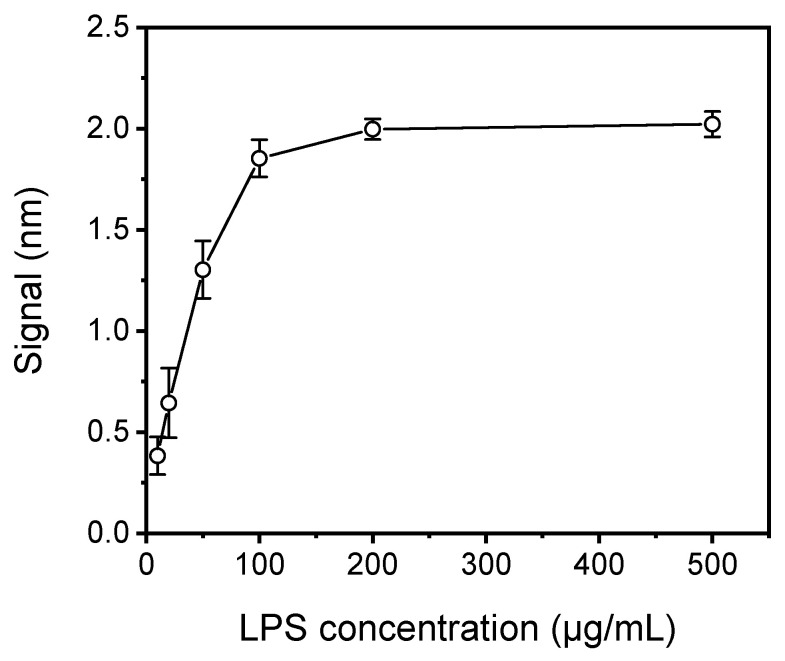
Plot of net mean signal values obtained for zero calibrator against *S. typhimurium* LPS concentration used for coating of the WLRS chips. Each point is the mean value of the signals obtained from five chips ± SD.

At first, the concentration of LPS used for coating the chips was optimized using chips spotted with LPS solutions with concentrations ranging from 10 to 500 μg/mL in coating buffer. All chips were assayed by running a 1:1 volume mixture of a 3 μg/mL anti-*Salmonella* antibody solution with assay buffer (zero calibrator) for 40 min. As shown in Figure 3, the signal increased and reached maximum plateau values at LPS concentrations higher than 100 μg/mL. In addition, for LPS concentrations ranging from 10 to 100 μg/mL the coefficients of variation (CVs) of responses obtained from five chips spotted with the same LPS concentration ranged from 26% to 15%, as opposed to CVs of approximately 3% obtained from chips spotted with LPS concentrations equal to or higher than 200 μg/mL. The rather high CV values observed for chips spotted with LPS concentrations lower than 200 μg/mL could be attributed to inadequate and heterogeneous coverage of the chip surface. Thus, a 200 μg/mL LPS solution was selected for further experimentation.

**Figure 4:**
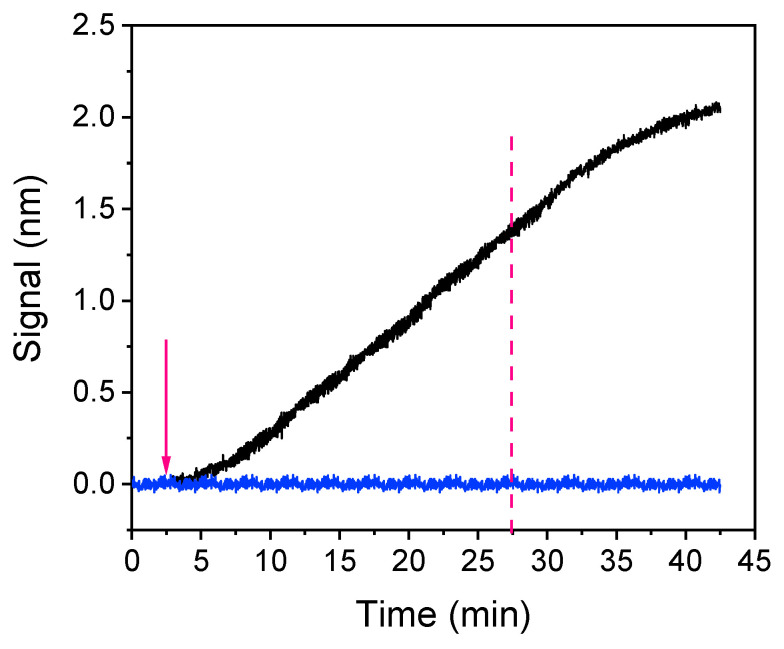
Real-time signal evolution upon passing over a chip coated with a 200 μg/mL *Salmonella* LPS solution zero calibrator consisting of a 3 μg/mL anti-*Salmonella* antibody solution mixed with assay buffer at a 1:1 volume ratio for 40 min (black line). For the assessment of non-specific binding, the zero calibrator signal obtained from a chip coated with bovine serum albumin (BSA) is indicated (blue line). The arrow indicates the time point of anti-*Salmonella* antibody solution introduction and the vertical, magenta dotted line the time point corresponding to 25 min after the introduction of the antibody solution.

The next parameter optimized was the assay duration. Figure 4 presents the real-time sensor response obtained for a zero calibrator, consisting of a 1:1 volume ratio of a 3 μg/mL rabbit polyclonal anti-*Salmonella* antibody solution with assay buffer, which ran for 40 min over a chip spotted with 200 μg/mL of *S. typhimurium* LPS. As shown, in order to achieve adequate signal (>1 nm), 25 min of immunoreaction were required (time point indicated by the vertical, magenta, dotted line in Figure 4) whereas the signal was continuously increasing even after 40 min of reaction. In addition to the specific immunoreaction signal (black line), the signal obtained from a chip spotted with BSA and assayed as the LPS-spotted chip is provided (blue line) to determine the non-specific binding signal. As shown, there was no measurable non-specific binding signal.

**Figure 5:**
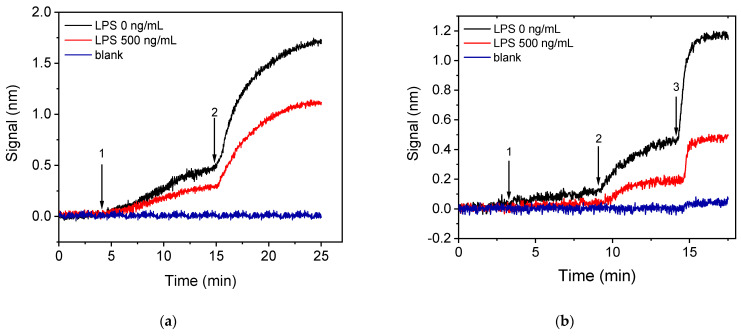
Real-time sensor response obtained from chips coated with a 200 μg/mL *Salmonella* LPS solution applying either the (**a**) two-step or (**b**) three-step assay configurations. The two-step assay configuration (**a**) included running over the chip 1:1 volume mixtures of calibrators with a 3 μg/mL rabbit anti-*Salmonella* antibody solution (arrow 1 to 2), followed by an Anti-Rabbit antibody solution (arrow 2 to end); while the three-step assay configuration (**b**) included running over the chip 1:1 volume mixtures of calibrators with a 1.5 μg/mL rabbit anti-*Salmonella* antibody solution (arrow 1 to 2), followed by biotinylated Anti-Rabbit IgG antibody (arrow 2 to 3) and streptavidin (arrow 3 to end). Black line corresponds to zero calibrator, red line to a calibrator containing 500 ng/mL LPS, and blue line to signal obtained from a chip spotted with BSA and assayed as the LPS-spotted chip.

In an attempt to minimize the assay duration and the anti-*Salmonella* antibody (primary antibody) consumption, a two-step assay configuration that included reaction with an Anti-Rabbit IgG antibody (secondary antibody) after the primary immunoreaction was investigated. In Figure 5a, the real-time sensor responses obtained for the zero calibrator and a calibrator containing 500 μg/mL of LPS, both mixed in a 1:1 volume ratio with a 3 μg/mL anti-*Salmonella* antibody solution run over the chip for 10 min and followed by a 10 μg/mL Anti-Rabbit IgG antibody solution for another 10 min, are presented. As shown, the signal achieved for the zero calibrator following the two-step assay format with a total assay duration of 20 min was 50% higher than the signal obtained from the primary immunoreaction at the same time interval. In addition, regarding the reaction with the secondary antibody, approximately 80% of the signal obtained after a 10 min reaction was obtained in the first 5 min. Thus, the duration of this step could be shortened to 5 min without significant reduction of the signal amplitude. Regarding the assay sensitivity, the percent signal for an LPS calibrator with a concentration of 500 ng/mL with respect to zero calibrator signal was approximately 65%. Since the assay sensitivity in a competitive assay is regulated by the primary antibody affinity for the analyte, a significant improvement of sensitivity can be achieved by considerably reducing the primary antibody amount used.

Thus, in order to decrease as possible both the primary immunoreaction duration and the primary antibody concentration a three-step assay configuration was investigated, involving two signal enhancement steps, i.e., reaction with biotinylated secondary antibody and streptavidin. It was found that, by employing a 10 min primary immunoreaction followed by a 5 min reaction with the biotinylated secondary antibody and a 3 min reaction with streptavidin, zero calibrator signals similar to those obtained with the two-step configuration, when employing a 5 min reaction with the non-biotinylated secondary antibody for the same primary immunoreaction duration, were obtained using a 1.5 μg/mL anti-*Salmonella* antibody solution instead of 3 μg/mL. Moreover, the primary immunoreaction duration would be reduced to 7 min without significant effect in the final signal. In Figure 5b, the real-time responses obtained for the zero calibrator and a calibrator containing 500 ng/mL LPS following the three-step configuration (7 min primary immunoreaction, 5 min biotinylated secondary, 3 min streptavidin) are provided. As it is shown, the zero calibrator signal was slightly reduced (20%) compared to the two-step assay, whereas the percent signal corresponding to the 500 ng/mL calibrator with respect to the zero calibrator signal was approximately 40%. This finding means that the three-step assay resulted in significant improvement in assay sensitivity, and, at the same time, the anti-*Salmonella* antibody consumption was reduced by more than 50% while the assay duration remained 15 min. Moreover, the non-specific binding signal, determined from a BSA-spotted chip, for both two-step and three-step assay configurations is provided in Figure 5a,b, respectively. As shown, there was a slight non-specific signal in the case of the three-step assay with accounted for less than 5% of the specific signal. For all these reasons, the three-step assay configuration employing reaction with biotinylated secondary antibody and streptavidin was selected for further experiments. The particular format enabled reduction of the whole assay cycle to 15 min instead of 25 min, required when only the primary immunoreaction was employed. In addition, the signal enhancement achieved by the two additional reaction steps allowed a 50% reduction of the primary antibody amount used resulting in improved assay sensitivity as was depicted in the increased signal drop obtained in presence of the analyte.

**Figure 6:**
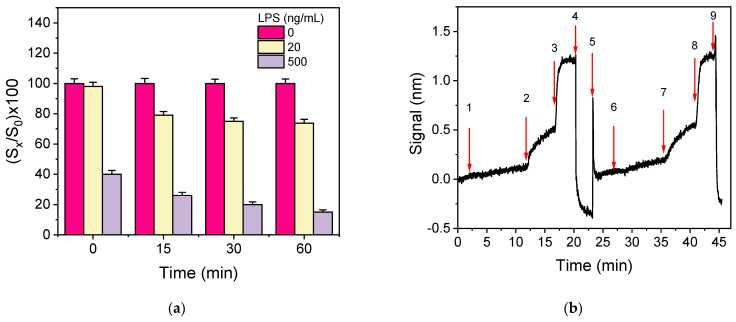
(**a**) Percent signal values obtained for the zero calibrator (magenta columns) or a calibrator containing 20 (yellow columns) or 500 ng/mL of LPS (grey columns) without or with 15, 30, and 60 min pre-incubation of the calibrators with the anti-*Salmonella* antibody solution (1.5 μg/mL). Each column represents the mean value of five measurements ± SD. (**b**) Real-time sensor response obtained upon running zero calibrator prepared in assay buffer (arrows 1–4), regeneration (arrows 4–6), and zero calibrator in bottled water (arrows 6–9).

Another approach to increase the detection sensitivity is to pre-incubate the calibrators/samples with antibody solution prior to the reaction with the immobilized analyte. This way, the reaction of the antibody with the analyte in the solution is favored over the reaction with the immobilized analyte, and higher percent signal drops could be obtained with respect to those received without pre-incubation. To test this possibility, mixtures of LPS calibrators (10 and 500 ng/mL) with the anti-*Salmonella* antibody were incubated for 5, 15, 30, and 60 min. The signals obtained when the pre-incubated mixtures were assayed were compared to the signal obtained from a mixture of zero calibrator with the antibody prepared and run immediately afterward. As shown in Figure 6a, pre-incubation for ≥15 min considerably improved the assay sensitivity since the percent signal obtained for the calibrator with the lower concentration (20 ng/mL), with respect to zero calibrator, dropped from 98% to 72%, whereas for the high-concentration calibrator (500 ng/mL), the percent signal dropped from 40% to 15%. Although the longer the duration of pre-incubation, the higher the percent signal drop, the improvement of percent signal drop for the lower concentration calibrator was marginal (approximately 10%) when the pre-incubation duration was increased from 15 to 60 min. Thus, 15 min pre-incubation was adopted in the final protocol.

In order to investigate the potential of *S. typhimurium* detection in drinking water samples with the immunosensor developed, as well as to select the optimum medium for the preparation of bacteria calibrators, the effect of water on the immunoassay performance was investigated. For this purpose, the calibration curves obtained from calibrators containing different concentrations of *S. typhimurium* bacteria prepared in tap or bottled water were compared to that received from calibrators prepared in assay buffer. It was found that the three resulting calibration curves were superimposed, indicating that tap and bottled water did not affect the assay sensitivity. In addition, as shown in Figure 6b, where the real-time signal obtained when sequentially running zero calibrator prepared in assay buffer and then, after regeneration, zero calibrator in bottled water are provided, identical zero calibrator signals were received in both matrices. Hence, the calibrators will be prepared in assay buffer.

**Figure 7:**
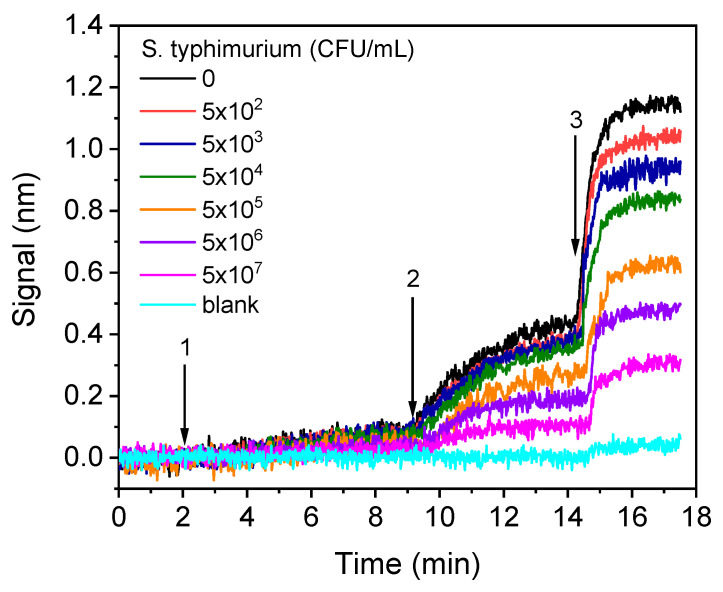
Real-time sensor responses corresponding to *S. typhimurium* bacteria calibrators with concentrations from 5 × 10^2^ to 5 × 10^7^ CFU/mL prepared in assay buffer. The arrows indicate the sequence of solutions running over the chip: assay buffer, start to arrow 1; mixture of calibrators with anti-*Salmonella* antibody, arrow 1–2; biotinylated anti-rabbit IgG antibody, arrow 2–3; streptavidin.

The real-time signal recordings provided from chips coated with LPS upon running calibrators with bacteria concentrations ranging from 5 × 10^2^ to 5 × 10^7^ CFU/mL are provided in Figure 7.

### 3.2. Analytical Characteristics

**Figure 8:**
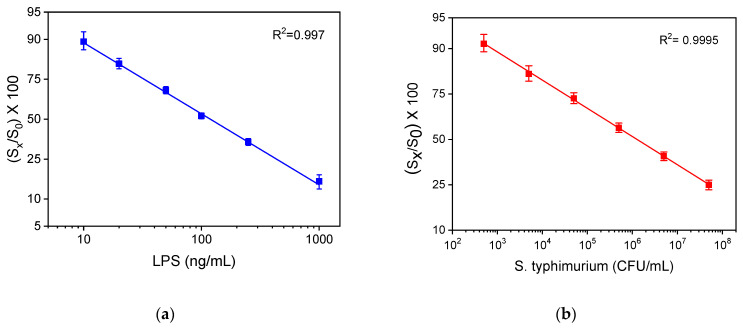
Typical calibration curves obtained for (**a**) LPS and (**b**) bacteria with the WLRS system applying the three-step assay configuration. Each point corresponds to the mean value of the signals obtained from three chips ± SD.

The calibration curves of LPS and *S. typhimurium* bacteria obtained with the WLRS sensor and the three-step assay, with a total assay time of 15 min, are presented in Figure 8a,b, respectively. The detection limit (LOD) of the assay was calculated as the concentration corresponding to signal that equals to the mean valueof 20 replicate measurements of zero calibrator -3SD, and it was determined to be 4 ng/mL for LPS and 320 CFU/mL for bacteria. In addition, the quantification limit (LOQ), which corresponds to the concentration of the mean value of 20 replicate measurements of zero calibrator -6SD, was found to be 10 ng/mL for LPS and 600 CFU/mL for bacteria. The dynamic range of the assay was 10–1000 ng/mL, and 600–5 × 10^7^ CFU/mL for LPS and bacteria, respectively.

**Figure 9:**
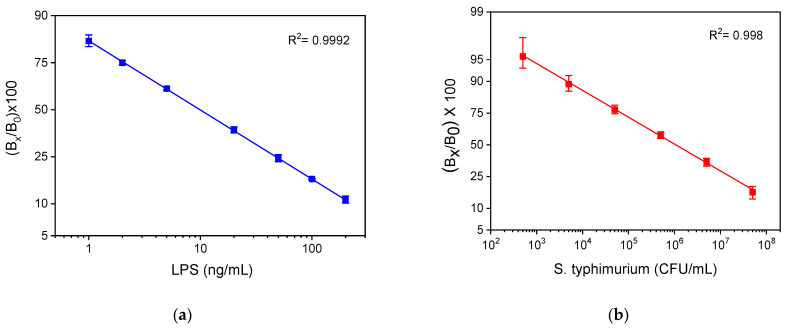
Typical calibration curves obtained for (**a**) LPS and (**b**) bacteria with the in-house developed competitive ELISA. Each point is the mean value of three measurements ± SD.

For comparison reasons, the calibration curves obtained for LRS and whole bacteria with the competitive ELISA developed in-house using the same immunoreagents are presented in Figure 9a,b, respectively. The detection limits of these assays were 0.4 ng/mL and 600 CFU/mL, respectively. Thus, regarding LPS, the LOD achieved with the WLRS sensor was 10 times higher than that obtained with the respective ELISA, while, regarding bacteria, the LOD obtained for both methods was the same. On the other hand, the duration of the WLRS assay was eight times shorter compared to that of ELISA, which was more than 2 h.

The assay repeatability was determined by assaying three control samples prepared in water spiked with three different concentrations of bacteria, i.e., 6 × 10^3^, 6 × 10^4^, and 6 × 10^5^ CFU/mL. The intra-assay coefficients of variation (CVs) were determined from four repetitive measurements of each control within the same day and ranged from 1.5% to 3.7%. The inter-assay CVs were calculated from five measurements carried out on five different days in a period of 1 month and varied from 3.1% to 7.5%.

The accuracy of the assay was evaluated through recovery experiments. For this purpose, tap and bottled water samples were spiked with three different concentrations of *S. typhimurium* (8 × 10^3^, 8 × 10^4^ and 8 × 10^5^ CFU/mL). The analysis of the samples was performed in triplicate prior to and after the addition of *S. typhimurium*, and the recovery values in percentage were calculated as the ratio of the bacteria concentration determined with respect to the concentration added in each sample. In Table 1, the mean values obtained from the four spiked samples along with recovery values in percentage are presented. The recovery values ranged between 92.5% to 108%, indicating the high accuracy of the determinations performed with the WLRS immunosensor.

The specificity of anti-*Salmonella* assay versus potential cross-reactants, such as *Salmonella thomson* and *E. coli* O157:H7, was tested through cross-reactivity experiments. *S. thomson* was selected since it was not amongst the *Salmonella* serotypes used as immunogens for the production of rabbit polyclonal antibody used in the study (see Section 2.1). *E. coli* was also used because it is one of the bacteria more frequently found in contaminated waters. Thus, *S. thomson* and *E. coli* cultures were used for the preparation of bacteria concentrations ranging from 10^4^–10^8^ CFU/mL and used as calibrators in the three-step *Salmonella* assay. Percent cross-reactivity (%CR) was determined using the equation

**Figure 10:**
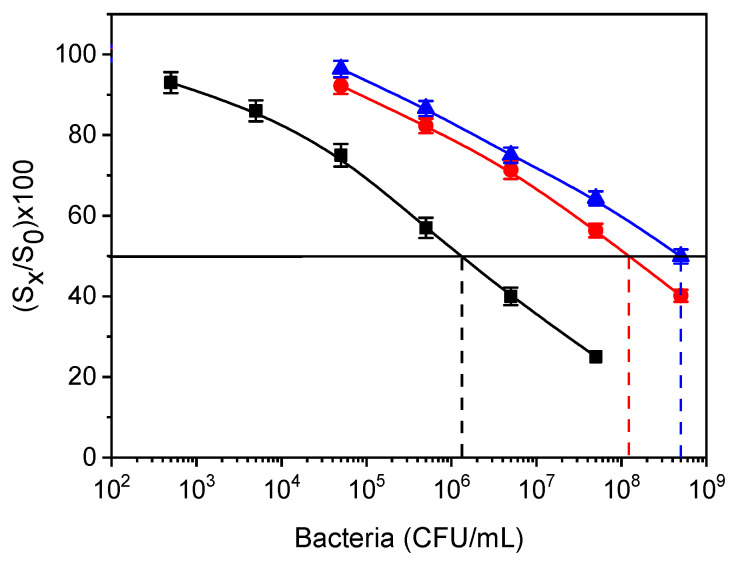
Calibration curves for *S. typhimurium* (black squares), *E. coli* (red circles), and *S. thomson* (blue triangles) obtained from LPS-coated WLRS chips. The dashed vertical lines indicate the bacteria concentration that corresponds to a 50% signal drop with respect to zero calibrator (horizontal black line). Each point is the mean value of the signals obtained from three chips ± SD.

%CR = (IC_50_*S. typhimurium*/IC_50_ cross-reactant bacterium) × 100 (1)

where IC_50_ *S. typhimurium* is defined as the concentration of *S. typhimurium* that provided a 50% signal drop, whereas IC_50_ cross-reactant bacterium is the concentration of the tested bacteria corresponding to a 50% signal drop with respect to zero calibrator. The cross-reactivity values determined were approximately 0.25% for *S. thomson* and 1.1% for *E. coli* (Figure 10). Thus, it can be concluded that the anti-*Salmonella* antibody and the respective assay were highly specific.

### 3.3. Regeneration

**Figure 11:**
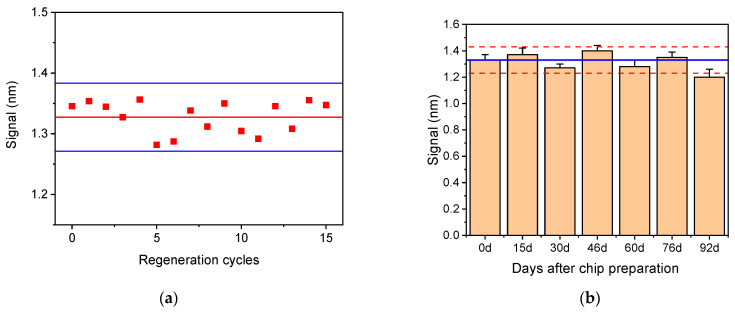
(**a**) Zero calibrator signal obtained from a single chip after 15 repetitive regeneration cycles. Horizontal solid red line corresponds to the mean value of 15 measurements, while the blue lines correspond to mean value ± 2SD. (**b**) Zero calibrator signals obtained from 21 chips modified with *Salmonella* LPS and tested over a period of 3 months. Each bar is the mean value of three chips ±SD. Blue line corresponds to mean value of the first six runs (up to day 76), and dashed red lines correspond to mean value ± 2SD.

In order to significantly reduce the analysis cost, the potential for chip regeneration was evaluated. For this purpose, several solutions such as 0.1 M glycine-HCl buffer, pH 2.5, 50 mM HCl, and 50 mM NaOH were tested to select the most effective solution for chip regeneration. Thus, after the completion of the total assay cycle, each of the regeneration solutions were pumped over the chip surface for 3 min, followed by a reaction with biotinylated Anti-Rabbit IgG antibody and streptavidin to determine the amount of primary antibody that remained after regeneration. It was found that the optimum solution for chip regeneration was 50 mM HCl, and it was selected for the final protocol. Furthermore, the potential of the chip to be reused after regeneration was tested by performing repetitive assay cycles with zero calibrator. As presented in Figure 11a, the chip could be regenerated at least 15 times without any signal loss. This finding is also proof that the LPS immobilized onto the chip through physical adsorption is strongly bound and does not desorb during the repetitive assay/regeneration cycles.

The storage stability of the LPS-modified chips was determined by preparing a batch of 21 chips and stored them in a desiccator at 4 °C for a period of three months. At regular time intervals, three chips were taken out and tested. In Figure 11b, the mean values obtained for the zero calibrator over the period of 3 months are provided. As shown, only a slight decrease (approximately 10%) in the zero calibrator signal value was observed after 3 months of storage, which, however, did not affect the calibration curve. Thus, it could be claimed that the LPS-modified chips are stable for at least 3 months under the specified storage conditions.

### 3.4. Comparison with Other Immunosensors

In Table 2, the LODs, assay time, and sample on which the detection of *S. typhimurium* was performed, employing label-free optical immunosensors published in the literature, are listed, along with the respective values of the developed WLRS immunosensor. As shown, the majority of the literature sensors are based on the surface plasmon resonance principle (SPR) [27,44,45,46,47,48,49,50,51]. In terms of *S. typhimurium* detection, the proposed immunosensor is at least 100 times more sensitive and four to six times faster than the reported SPR sensors. An Ω-shaped fiber-optic localized SPR sensor for the detection of *S. typhimurium* was also reported with an LOD approximately three times lower than the LOD achieved with the developed WLRS sensor [26]. However, the analysis time of the proposed immunosensor was six times lower compared to that of the Ω-shaped fiber-optic localized SPR sensor. In addition, a fiber-optic immunosensor with an LOD of 247 CFU/mL could detect *S. typhimurium* in milk with assay time similar to that of the developed WLRS sensor [52], while an optical grating based sensor provided a 4-time higher LOD for an assay duration of 10 min [53]. An immunosensor based on Hartman interferometry capable to detect *S. typhimurium* in 10 min has been reported in the literature, with an LOD 30 times higher than that provided by the proposed immunosensor [36]. In addition, two sensors based on surface-enhanced Raman spectroscopy (SERS) have been also reported for *Salmonella typhimurium* detection [54,55]. The first one had approximately 3 × 10^5^ times higher LOD than the proposed sensor, and the assay duration was not mentioned, whereas the second one achieved three-times lower LOD compared to the proposed sensor employing, however, a fivefold longer assay procedure. Finally, a label-free fluorescent aptasensor based on the fluorescence energy transfer (FRET) principle was developed with an LOD of 733 CFU/mL achieved with a 2 h assay [56]. Overall, the immunosensor developed is one of the faster and more sensitive label-free optical sensors reported in the literature. From the detection limits presented in Table 2, it is obvious that none of the label-free optical sensors has the required sensitivity to meet the requirements for direct, on-site determination of *S. typhimurium* in water or food samples. In all cases, either a pre-concentration step from high sample volumes, e.g., by water filtration or immunomagnetic concentration of bacteria cells or a pre-enrichment step to increase the concentration of bacteria present in the sample is needed. Taking into account that *Salmonella* cells replicate themselves every 40 min, it would take approximately 8 h for a single, viable cell per 25 mL to create a population of approximately 600 CFU/mL, which is the LOQ of our method.

## 4. Conclusions

A WLRS biosensor for the detection of *Salmonella* LPS and bacteria in drinking water samples was presented for the first time. The WLRS system allowed for label-free and real-time detection of bacteria employing a three-step assay configuration in 15 min. The analytical performance of the immunosensor was characterized by high sensitivity, accuracy, and reproducibility. The WLRS biosensor provides similar sensitivity with the in-house ELISA method, which was completed in more than 2 h. In addition, the WLRS biochip could be regenerated and reused at least 15 times, thus significantly reducing the analysis cost. Given the detection limit achieved, the short analysis time, and the small size of the chip, the proposed immunosensor could find wide application for bacteria detection in drinking water. Moreover, taking into account that fast bacteria detection is of high importance for the food industry since it could considerably reduce the time interval between production and release of the products, the developed immunosensor could facilitate the quality assessment process across production lines. In addition, since the proposed WLRS sensor is an analytical platform, it could find application in the detection of other bacteria by functionalizing the chips with appropriate antigens or specific antibodies.

## Figures and Tables

**Table 1:** Recovery of known amounts of *S. typhimurium* bacteria spiked in four different water samples, including tap water and three bottled Greek natural mineral water products (Vikos, Zagori, and Avra).

Sample	Amount Added (CFU/mL)	Amount Determined (CFU/mL)	% Recovery
Tap water	8 × 10^3^	8.2 × 10^3^	102
	8 × 10^4^	7.9 × 10^4^	98.7
	8 × 10^5^	7.6 × 10^5^	95.0
Bottled water (Vikos)	8 × 10^3^	8.6 × 10^3^	108
	8 × 10^4^	7.6 × 10^4^	95.0
	8 × 10^5^	8.2 × 10^5^	102
Bottled water (Zagori)	8 × 10^3^	7.6 × 10^3^	95.0
	8 × 10^4^	8.1 × 10^4^	101
	8 × 10^5^	7.5 × 10^5^	93.8
Bottled water (Avra)	8 × 10^3^	7.4 × 10^3^	92.5
	8 × 10^4^	7.7 × 10^4^	96.2
	8 × 10^5^	8.4 × 10^5^	105

**Table 2:** Comparison of the developed WLRS immunosensor with other studied optical label-free immunosensors for the detection of *S. typhimurium*.

Immunosensor/Device	Sample Type	LOD (CFU/mL)	Assay Time (min)	Ref. #
SPR	romaine lettuce	4.7 × 10^5^	<6	[27]
SPR	milk	2.5 × 10^5^	~100	[44]
SPR	chicken carcass	1 × 10^6^	~17	[45]
Portable SPR	buffer	10^7^	~60	[46]
SPR	buffer	1.7 × 10^3^	22	[47]
SPR	assay buffer	10^6^	≤120	[48]
SPR	buffer	10^5^	10	[49]
SPR	buffer	-	6–7	[50]
SPR imaging	buffer chicken carcass rinse	2.1 × 10^6^ 7.6 × 10^6^	20	[51]
Ω-shaped fiber-optic LSPR	buffer	<128	100	[26]
Fiber-optic	milk	247	<20	[52]
Hartman interferometry	assay buffer chicken carcass	10^4^	10	[36]
Optical-grating coupler	buffer	1.3 × 10^3^	60	[53]
SERS	buffer	10^8^	-	[54]
SERS	buffer	100	75	[55]
FRET aptasensor	buffer	733	120	[56]
WLRS immunosensor developed	tap and bottled water	320	15	

## Data Availability

The data presented in this study are available on request from the corresponding author. The data are not publicly available due to privacy issues.
